# Influence of the socioeconomic status on the prevalence of malocclusion in the primary dentition

**DOI:** 10.1590/2176-9451.20.1.074-078.oar

**Published:** 2015

**Authors:** Thiene Silva Normando, Regina Fátima Feio Barroso, David Normando

**Affiliations:** 1 Master's student in Dentistry, Federal University of Pará (UFPA); 2 Associate professor, UFPA; 3 Adjunct professor, UFPA

**Keywords:** Malocclusion, Primary dentition, Socioeconomic factors

## Abstract

**OBJECTIVE:**

To assess the influence of socioeconomic background on malocclusion prevalence in primary dentition in a population from the Brazilian Amazon.

**METHODS:**

This cross-sectional study comprised 652 children (males and females) aged between 3 to 6 years old. Subjects were enrolled in private preschools (higher socioeconomic status - HSS, n = 312) or public preschools (lower socioeconomic status - LSS, n = 340) in Belém, Pará, Brazil. Chi-square and binomial statistics were used to assess differences between both socioeconomic groups, with significance level set at P < 0.05.

**RESULTS:**

A high prevalence of malocclusion (81.44%) was found in the sample. LSS females exhibited significantly lower prevalence (72.1%) in comparison to HSS females (84.7%), particularly with regard to Class II (P < 0.0001), posterior crossbite (P = 0.006), increased overbite (P = 0.005) and overjet (P < 0.0001). Overall, malocclusion prevalence was similar between HSS and LSS male children (P = 0.36). Early loss of primary teeth was significantly more prevalent in the LSS group (20.9%) in comparison to children in the HSS group (0.9%), for both males and females (P < 0.0001).

**CONCLUSION:**

Socioeconomic background influences the occurrence of malocclusion in the primary dentition. In the largest metropolitan area of the Amazon, one in every five LSS children has lost at least one primary tooth before the age of seven.

## Abstract (pt)

**OBJETIVO:**

avaliar a influência da condição socioeconômica na prevalência de má oclusão na dentição decídua em uma população amazônica.

**MÉTODOS:**

esse estudo transversal compreendeu 652 crianças, de ambos os sexos, entre 3 e 6 anos de idade. Os indivíduos estavam matriculados na pré-escola na rede privada de ensino (alto nível socioeconômico; n = 312) ou, rede pública (baixo nível socioeconômico; n = 340), em Belém, no Pará. O teste chi-quadrado e estatística binominal foram usados para avaliar as diferenças entre os grupos socioeconômicos, com nível de significância considerado em p < 0,05.

**RESULTADOS:**

foi observada uma alta prevalência de má oclusão (81,44%) na amostra examinada. As meninas das escolas públicas exibiram uma prevalência significativamente menor (72,1%) em comparação às das escolas privadas (84,7%), principalmente com relação à prevalência da má oclusão de Classe II (p < 0,0001), mordida cruzada posterior (p = 0,006), sobremordida (p = 0,005) e sobressaliência (p < 0,0001). De maneira geral, a prevalência de má oclusão foi similar entre as crianças do sexo masculino dos dois grupos (p = 0,36). A perda precoce de dente decíduo foi significativamente mais prevalente no grupo com menor nível socioeconômico (20,9%) quando comparada à de crianças nas escolas privadas (0.9%), em ambos os sexos (p < 0,0001).

**CONCLUSÃO:**

a condição socioeconômica influencia a ocorrência de má oclusão na dentição decídua. Na maior metrópole da Amazônia, uma em cada cinco crianças do grupo com baixo nível socioeconômico perdeu, no mínimo, um dente decíduo antes dos sete anos.

## INTRODUCTION

Literature reveals the prevalence of malocclusion in approximately 80-90% of Brazilian children in mixed and permanent dentition,^1^^-^ 5 while these data are conflicting for the primary dentition, ranging from 50% to 80%.^6^^-^ 12 This discrepancy may be explained by the various diagnostic criteria employed,^9^ the examiner's background, as well as research subjects' age and ethnicity. Additionally, the socioeconomic background of the studied population seems to be another relevant etiologic factor.^3^ Despite the large number of epidemiologic investigations regarding malocclusion prevalence, only a few have examined the etiologic factors that could be controlled by health measures.^13^

Brazil has one of the steepest socioeconomic disparities around the world.^14^ Only a few studies have investigated the influence of this variable on malocclusion prevalence. The main difficulty appears to be the access to the *most economically privileged* groups, particularly because they usually refuse to participate in this kind of investigation.^7^^,^ 9 Data on primary dentition in children are contradictory and restricted to cities in São Paulo state, the wealthiest Brazilian state, in which socioeconomic imbalance is not as evident as in other regions. Studies conducted in São Paulo have not identified any influence of patients' socioeconomic background on malocclusion,^7 ^except for a study conducted in Bauru city (São Paulo state),^9^ which reported a higher risk of malocclusion prevalence in children studying in public schools (lower socioeconomic status).

A limited number of studies have investigated the socioeconomic influence on malocclusion prevalence in populations in primary dentition around the world. In developed countries,no significant influence of patients' socioeconomic background has been identified.^15^^,^ 16 However, in underdeveloped areas, a significantly higher prevalence of posterior crossbite is observed among children belonging to higher socioeconomic classes.^17^ Moreover, children with lower socioeconomic status (LSS) had significantly greater early primary tooth loss.

Cities located in the Brazilian Amazon are characterized by *trihybrid* ethnic miscegenation involving Latin/European Caucasians, Brazilian African-descendants, and Amerindians.^18^ Furthermore, there is a clear socioeconomic imbalance among people living in this region. Determining the actual prevalence of malocclusion in such a population should elucidate the influence of socioeconomic status on malocclusion.

## MATERIAL AND METHODS

A random selection of children were examined, males and females, with *complete primary dentition. Subjects ranged in age* from 3 to 6 years old, and were enrolled in preschools in Belém (Pará, Brazil), city with mean population of 1.5 million. 

In general, the richer population in Brazil attends private schools, while the poor one attends public schools.^19 ^This criterion was used to assess the groups of the present study. The sample was divided into two groups: The first one comprising children attending public preschools (representing children from a lower socioeconomic status), and the second group comprising children attending private preschools (representing children from a higher socioeconomic status). Comparative analysis between groups was carried out to assess potential differences in malocclusion prevalence.

Sample size calculation was carried out on the basis of the school population data obtained from the Municipal Secretary of Education. In total, 14,356 children were enrolled in Belém kindergarten schools: 6,898 of which were enrolled in private schools whereas 7,458 were enrolled in public schools. A value of P = 0.5 was used for sample size calculation (estimated malocclusion prevalence),^6^ confidence *level at* 95% and sample error of 4%. These calculations indicated that it would be necessary a sample comprising 576 children, 276 from private schools and 300 from public schools.

This study was submitted to the Federal University of Pará Ethics Committee on Human Research, and approved under protocol #143/06 CEP-ICS/UFPA. The study was also approved by the kindergarten coordination staff and children's parents. Students were selected to represent the entire metropolitan area encompassed by the city of Belém, Pará, located in the Brazilian Amazon region.

The schools selected were the ones which presented the highest number of children enrolled in the age range established for this study and which also allowed this research to be conducted. Four public schools (n = 4) were selected. All of them were located at the city suburb (the poorest area).

The private schools (n = 5) were located in the central area of Belém (the wealthiest area). The sample was collected at the schools approving the collection of data, since the access with scientific research purposes is frequently denied by Brazilian private schools. 

Children's clinical examinations were performed by one single specialist in Orthodontics. Overjet, overbite, transverse dental arch relationship, early tooth loss, and occlusal relationship (Class I, II and III) were all examined. The normal characteristics of dentition were assessed as described in the literature.^6^^,^ 9

Inter-group comparative analysis was performed by means of chi-square test. Binomial test was used to compare the populations of public and private schools with regard to different morphological types of malocclusion. The level of confidence in all statistical analyses was 95% (P < 0.05). Reproducibility of clinical examination was verified by means of Kappa's statistics.

## RESULTS

To assess reproducibility of clinical examination, Kappa test was applied in 8.6% (n = 56) of the sample. Results revealed satisfactory reproducibility (Kappa = 0.72, P < 0.01). 

The prevalence of malocclusion found in this study was 81.44% (Table 1). Class II malocclusion was the most prevalent, occurring in 67.5% of cases. Class I malocclusion was observed in 9.4% of children, whereas Class III prevalence was observed among 4.5% of cases (Table 1).

**Table 1 -:** Frequency distribution of children enrolled in public preschools (lower socioeconomic status; LSS) or private preschools (higher socioeconomic status; HSS), according to the sex and malocclusion characteristics.

	Male (n = 341)		P-value (Male)	Female (n = 311)		P-value (Female)	LSS (n = 340)	HSS (n = 312)	Odds ratio Male + Female	Total (n = 652)
	LSS (n = 186)	HSS (n = 155)	LSS x HSS	LSS (n = 154)	HSS (n = 157)	LSS X HSS	Male + Fem	Male + Fem	LSS X HSS	Male+ Fem
Normal x Malocclusion										
Normal	33 (17.7%)	21 (13.5%)	0.36 (ns)	43 (27.9%)	24 (15.3%)	0.01**	76 (22.4%)	45 (14.4%)	0.59* (0.4-0.9)	121 (18.6%)
Malocclusion	153 (82.3%)	134(86.5%)		111 (72.1%)	133 (84.7%)		264 (77.6%)	267 (85.6%)		531 (81.4%)
Malocclusion classification										
Class I	18 (9.7%)	16 (10.3%)	0.12 (ns)	20 (13.1%)	7 (4.5%)	0.013*	38 (11.2%)	23 (7.4%)		61 (9.4%)
Class II	121 (65.1%)	114 (73.6%)		80 (51.9%)	125 (79.6%)	< 0.0001***	201 (59.1%)	239 (76.6%)		440 (67.5%)
Class III	14 (7.5%)	4 (2.6%)		11 (7.1%)	1 (0.6%)	0.007**	25 (7.3%)	5 (1.6%)		30 (4.5%)
Malocclusion type										
Overbite >	38 (20.4%)	46 (29.7%)	0.048*	23 (14.9%)	44 (28%)	0.005**	61 (17.9%)	90 (28.8%)	0.54* (0.4-0.8)	151 (23.2%)
Open bite	12 (6.5%)	8 (11.6%)	0.09 (ns)	11 (7.1%)	18 (11.5%)	0.19 (ns)	23 (6.8%)	26 (8.3%)	0.80 (0.5-1.4)	49 (7.5%)
Overjet >	27 (14.5%)	25 (16.1%)	0.68 (ns)	8 (5.2%)	27 (17.2%)	< 0.0001***	35 (10.2%)	52 (16.7%)	0.57* (0.4-0.9)	87 (13.3%)
Anterior crossbite	12 (6.5%)	9 (5.8%)	0.80 (ns)	7 (4.5%)	2 (1.3%)	0.09 (ns)	19 (5.6%)	11 (3.52%)	1.62 (0.8-3.5)	30 (4.6%)
Posterior crossbite	7 (3.8%)	15 (9.7%)	0.026 *	3 (1.9%)	14 (8.9%)	0.006**	10 (2.9%)	29 (9.3%)	0.30* (0.1-0.6)	39 (6.0%)
Early tooth loss	40 (21.5%)	3 (1.9%)	< 0.0001***	31 (20.1%)	0 (0%)	< 0.0001***	71 (20.9%)	3 (1.0%)	27.2** (8.5-87.3)	74 (11.3%)

(ns)= not significant;

* P < 0.05;

** P < 0.01;

*** P < 0.001.

Regarding the influence of socioeconomic factors on malocclusion prevalence, the overall findings revealed that high socioeconomic status (HSS) children showed higher prevalence of malocclusion (OR=0.59, 95% CI= 0.39-0.88,Table 1). Class II malocclusion, overbite and increased overjet were less prevalent among female children in public preschools, as compared to their counterparts in private preschools (P < 0.0001,Table 1). With regard to male children, we did not observe any significant differences in the prevalence of malocclusion, when represented by the sagittal canine relationship (P = 0.12,Table 1). Nevertheless, increased overbite was significantly more frequent in the lower level socioeconomic group (P = 0.048).

When the various types of morphological malocclusion were analyzed, overbite was observed in 23.15% of the total sample. On the other hand, anterior open bite was observed in only 7.5% of children, with no significant influence of socioeconomic status (Table 1).

Anterior crossbite appeared in a very similar manner in both socioeconomic status, for both males (P = 0.085) and females (P = 0.805) (Table 1), with relatively low occurrence (4.60%). However, posterior crossbite presented higher prevalence among children attending private schools (HSS), for both males and females (P = 0.006; and P = 0.026,Table 1). Posterior crossbite was observed in 6% of the sample.

Early primary tooth loss was present in less than 1% of high socioeconomic status children and in 21% of low socioeconomic status children (OR = 27.19, 95% CI = 8.46 - 87.31). 

## DISCUSSION

Malocclusion prevalence in primary dentition observed in the present investigation (81.44%) is one of the highest reported in the literature.^6^-^12^,^21^ This high prevalence of malocclusion may be related to the high level of genetic miscegenation in Brazilian Amazon cities.^8^ This matter should be addressed in future investigations.

Results revealed that high socioeconomic status (HSS) children showed a higher prevalence of malocclusion. These data conflict with most previous studies on the same topic,^7^^,^ 15 ^,^ 16 which did not report any significant influence of socioeconomic background. Further investigations should explore environmental factors associated with socioeconomic background, such as sucking habits and breast-feeding,^13^^,^ 22 ^,^ 23 and their impact on the development of malocclusion.

One single study performed in a Venezuelan school^17^ found that children from public schools had a considerably higher prevalence of primary tooth loss, while posterior crossbite was significantly more prevalent among children from private preschools (high socioeconomic status). On the other hand, another study performed on a population of Brazilian children^9^ found a higher prevalence of anterior open bite and deleterious oral habits in children belonging to lower socioeconomic status. The present findings corroborate the findings obtained in Venezuela,^17^ since a significantly lower frequency of posterior crossbite (OR = 0.3, 95% CI = 0.14-0.62) and a significantly higher frequency of early tooth loss were observed in the lower socioeconomic group of children (OR = 27.19, 95% CI = 8.47-87.1). However, open bite does not seem to be related to socioeconomic background.

The reduced frequency of Class II and related malocclusions (overbite and increased overjet) among LSS female children is an interesting finding that cannot be explained by the data obtained herein. This tendency was also observed for male children, but was only significant in the case of overbite. Factors related to deleterious oral health must be investigated. A previous report^2^ stated that, while female children whose mothers had formal jobs had a significantly higher frequency of sucking habits, while no significant effect was observed for the male group.

Overbite was observed in one of every four children. This prevalence is higher than what was reported by previously published reports.^11^^,^ 24 On the other hand, anterior open bite was observed in only 7.5% of children, with no significant influence of socioeconomic status (Table 1). This open bite frequency seems to be lower than it is in previously published data^13^^,^ 22. The lower frequency of open bite and the higher level of overbite may be linked to the facial skeletal characteristics of the Amazon population, mainly the striking indigenous influence over the formation of urban populations.^18^

Anterior crossbite prevalence was similar in previous data.^6^^,^ 9 ^,^ 11 However, posterior crossbite was more prevalent among children attending private preschools (HSS), for both males and females (P = 0.006; and P = 0.026,Table 1). Posterior crossbite was observed in 6% of the examined sample, lower than typically reported.^6^^,^ 9 ^-^ 12 The fact that posterior crossbite is directly related to the presence of deleterious oral habits^9^^,^ 22 ^,^ 25 suggests that private preschool children are more likely to present these habits while breathing or sucking. Once again, this matter should be investigated in further studies, since there was no difference regarding anterior open bite between LSS and HSS groups. Non-breast-fed children presented significantly greater chances of having anterior open bite^26^ and posterior crossbite^27^ when compared with those who were breast-fed for periods longer than 12 months.

Literature describes lower prevalence of early primary tooth loss in other Brazilian regions.^9^^,^ 24 The present findings showed that one in every five LSS children in the largest Brazilian Amazon city had early loss of at least one primary tooth before the age of seven. The reasons for the trends outlined herein include lack of access by this part of the population to basic dental services, as well as deficient oral hygiene and a deficient public health system. The National Brazilian Social Project 2003^28^ showed that children in the northern region of the country present the highest frequency of untreated tooth decay. The DMF was approximately 27% higher in this region than in the southeast of Brazil, the wealthiest area. A longitudinal study^29^ revealed that children with loss of primary molars before 7 1/2 years old developed more crowding than children without losses or with losses after 7 1/2 years of age. Thus, the high incidence of primary tooth loss among children belonging to lower socioeconomic groups set a worrying picture for the future. It is expected that the current high prevalence of malocclusion undergoes even greater increase. 

## CONCLUSION

Malocclusion is highly prevalent in the primary dentition of urban Brazilian Amazon children. The influence of socioeconomic background on the prevalence of malocclusion varies according to the morphological classification of malocclusion. Class II, increased overbite and overjet, as well as posterior crossbite were significantly more frequent among children at a higher socioeconomic status. Furthermore, in Belém, the largest city in Brazilian Amazon, one in every five LSS children has lost at least one primary tooth before the age of seven. These results indicate the need for oral health policies that include preventive care so as to improve the dental health of this segment of the population.
